# Development of a Linear Acoustic Array for Aero-Acoustic Quantification of Camber-Bladed Vertical Axis Wind Turbine

**DOI:** 10.3390/s20205954

**Published:** 2020-10-21

**Authors:** Abdul Hadi Butt, Bilal Akbar, Jawad Aslam, Naveed Akram, Manzoore Elahi M Soudagar, Fausto Pedro García Márquez, Md. Yamin Younis, Emad Uddin

**Affiliations:** 1 Department of Mechanical Engineering, School of Mechanical and Manufacturing Engineering (SMME), National University of Science and Technology, Islamabad 44000, Pakistan; hadi_ul_lisan@yahoo.com (A.H.B.); jawadaslam@smme.nust.edu.pk (J.A.); emaduddin@smme.nust.edu.pk (E.U.); 2 Department of Mechanical Engineering, Mirpur University of Science and Technology (MUST), Mirpur 10250, AJK, Pakistan; bilal.akbar@must.edu.pk (B.A.); naveed.me@must.edu.pk (N.A.); myyounis.me@must.edu.pk (M.Y.Y.); 3 Department of Mechanical Engineering, Faculty of Mechanical Engineering, University of Malaya, Kuala Lumpur 50603, Malaysia; manzoor@siswa.um.edu.my; 4 Ingenium Research Group, University of Castilla-La Mancha, 45071 Ciudad Real, Spain

**Keywords:** aeroacoustic, tip speed ratio, blade passing frequency, anechoic chamber, dynamic stall

## Abstract

Vertical axis wind turbines (VAWT) are a source of renewable energy and are used for both industrial and domestic purposes. The study of noise characteristics of a VAWT is an important performance parameter for the turbine. This study focuses on the development of a linear microphone array and measuring acoustic signals on a cambered five-bladed 45 W VAWT in an anechoic chamber at different tip speed ratios. The sound pressure level spectrum of VAWT shows that tonal noises such as blade passing frequencies dominate at lower frequencies whereas broadband noise corresponds to all audible ranges of frequencies. This study shows that the major portion of noise from the source is dominated by aerodynamic noises generated due to vortex generation and trailing edge serrations. The research also predicts that dynamic stall is evident in the lower Tip speed ratio (TSR) region making smaller TSR values unsuitable for a quiet VAWT. This paper compares the results of linear aeroacoustic array with a 128-MEMS acoustic camera with higher resolution. The study depicts a 3 dB margin between two systems at lower TSR values. The research approves the usage of the 8 mic linear array for small radius rotary machinery considering the results comparison with a NORSONIC camera and its resolution. These observations serve as a basis for noise reduction and blade optimization techniques.

## 1. Introduction

The effect of increasing global warming has urged the world to adopt clean and green energy sources [1]. During the past decade, the most efficient and reliable clean energy source has been wind energy [2,3]. Wind energy generation is linked to wind turbines which offer efficiency and adaptability for domestic purposes as well. Vertical axis wind turbines (VAWT) are the most popular wind turbines for domestic-urban purposes [4]. However, the aerodynamic noise generated by blades of these VAWTs has become problematic for safe and quiet operations in public areas. In many cases of domestic use, it has been noted that the aerodynamic and mechanical noise has been a source of disturbance and has been reported as problematic in the neighborhood [5]. Throughout the years, wind farms have always been a concern to the nearby residents, becoming an obstacle to the boom of wind energy. People are worried about noise, whether it’s a night club, shopping mall or surrounding area [6]. Moreover, noise generation depicts a loss in performance and, therefore, it must be catered for. The complex terrains also cause difficulties in designing the layout of wind farms generating noise and thus causing sleeping issues to the residents since they require a relatively quieter environment [7]. One more negative effect of the noise generated from wind turbines is that it breaks the environmental balance by diverting the routes of migratory birds [8]. Legislation in different countries has also been developed to cater for wind turbines causing an environmental imbalance [9]. Therefore, the environmental and social acceptance of wind turbines is necessary for the green energy revolution, therefore the quantification and attenuation of the noise generated by wind turbine is of prime importance. Noise is generated in two different ways by wind turbines, i.e., mechanical noise and aerodynamic noise [10,11,12]. Mechanical noise is mainly produced by the bearings, gearbox and ground support [13,14], whereas the aerodynamic noise being generated from the rotor blade can be differentiated in three sections of the Ffowcs Williams–Hawking (FW–H) equation [15].

The Ffowcs Williams–Hawking equation can be scrutinized in three different parts: noise related to thickness source, noise related to aerodynamic loading and noise related to nonlinear flow. The primary concern in this study is the dominant source, i.e., the VAWT rotor and it operates at a relatively low speed, and hence the thickness does not play a vital role in noise at this level. Therefore, the thickness term by Williams is neglected and the blade’s noise is determined by the two main sources, i.e., aerodynamic loading and broadband range. Aerodynamic loading noise has a constraint of geometric change which is very critical in small-scale VAWTs; therefore, the main focus of this research is alleviating the broadband surface noise which is predominant in aeroacoustic sources [16]. Moreover, the aerodynamic loading causes the hydrodynamic fluctuation of vortices which create the acoustic waves propagating in the far field. Brian [17] showed that changing the solidity of the blades can have a massive effect on the fluttering range of the blade and it can suppress the noise up to 7 dBs. A stiffened fiberglass blade can be much quieter as compared to a non-stiffened blade [17]. One more important source is the low frequency source from 1–20 kHz that covers the infrared spectrum. It has to be quantified and studied because if the VAWT lies is close vicinity to a person’s ear than this particular range can have vibroacoustic effects like fatigue, light headedness and nausea [18]. Dratva concluded that these high and low frequency noises have proved to cause severe implications in vulnerable communities such as adults with high blood pressures, diabetes etc. [19]. 

The broadband sources in blades also termed as airfoil self-noise, can further be classified in five different categories, i.e., turbulent boundary layer noise; trailing edge noise; stall noise; vortex shedding noise; tip vortex formation noise, and; laminar vortex shedding noise [20], These five sources are related to flow interaction with the trailing edge. In almost all VAWTs, broadband noise at the trailing edge is dominant over noise at the leading edge because atmospheric turbulence is minimal as compared to boundary layer turbulence. Therefore, modification of the trailing edge with bioinspired serration is a good solution for attenuation of noise sources [21]. The dominant peaks in the low-frequency region as referred to by Ffowcs Williams and Hawking refer to tonal noises such as blade passing frequencies. In the broadband spectrum the strongest source of noise due to unsteady loading is the noise mitigated by dynamic stall occurring at low tip speed ratio (TSR) [22]. This problem has been addressed by optimization of a critical TSR value and providing a preset pitch angle to the blades [23]. 

As discussed above, trailing edge noise is a part of the self-generated aerofoil noises, and this noise is stimulated when the trailing edge is encountered with turbulent flow passed through the blade. This interface generates a broadband noise which is a dominant source in horizontal axis wind turbine (HAWT) and VAWT [20]. These sources have been modeled numerically using FW–H equations. Ghasemian and Nejat [24] performed numerical simulations to calculate aerodynamic noise propagated from a VAWT by using the large eddy simulation (LES) method for unsteady flows and FW-H acoustic analogy at five different TSR values. They depicted the trend of increasing sound pressure level is proportional to the revolutions per minute of rotating body and the tip speed ratio. Moreover, the SPL values vary with a logarithmic trend as we move away from the noise source. Wasala [25] predicted the far-field spectrum using large eddy (L-E) simulations, where the HAWT rotor produces the maximum noise. An experimental CART-2 HAWT was compared with these results for technical agreement. 

There have been different methods developed over time for the noise source characterization of VAWTs using computational methods and the experimental characterization using symmetrical airfoils. The camber in airfoil, incidence angle, solidity and serrations also have a significant amount of effect on noise generation in gusts [26]. Mohamed [15] did research on a Darrius wind turbine using the Ffowcs-Williams and Hawking numerical method and varied the TSR, rigidity and solidity. The OASPL of the turbine decreased by 7.6 dB after reducing the stiffness from 0.25 to 0.1. Moreover, the noise reduction between source and sink was 5.86 dB per unit and a similar study has also been carried out in this research [15].

The first step, before the application of noise reduction techniques, is identification, quantification, and segregation of dominant noise sources in a VAWT configuration. In order to track the dominant sources, microphone arrays are widely used. Different types of advanced acoustic quantification devices have been produced during the last three decades. In order to make long time measurements, different types of acquisition setups, mostly high-end portable arrays, have been developed over the years to acquire noise at different conditions since field measurements are difficult to register for all environmental conditions [27].The prime function of these devices is that they enhance the omnidirectional response of a microphone to a multidirectional response. The resolution of an array is enhanced when the span of array is larger covering broader area. Ranging from aircraft to high speed trains, microphone arrays have proved their efficacy in locating various noise sources. A microphone array comprises of a certain number of microphones placed in either linear, square, circular, or spiral orientation depending upon their directivity [28]. Amplitude and phase signals are collected from this array, and a spectrum is displayed which gives the sound level of the source and its directivity. An advantage of developing a microphone array is to incorporate the ability to achieve a directivity pattern in its signal reception. Delay and sum beamforming are two of the processes of steering the array’s direction automatically toward a chosen direction of arrival (DOA) or a specific point in space. The type, size, and shape of an array and postprocessing algorithm determines the accuracy of spectral analysis [29]. 

The microphone array can be utilized to quantify the noise sources, and also provide the directivity, since for the quantification of the sources the algorithm used for SPL use the RMS values. It does not have to calculate the phase difference for this term [30]. However, if the array is equipped with large number of high-end MEMS or microphones, the phase difference between each microphone can be calculated and used to find the 2D or 3D direction of arrival using cross spectra [31]. Blanchard et al. [31] have estimated the source localization of a unmanned aerial vehicles (UAV) using this method assuming two peaks one for reference signal and one for the calculation. The peaks are pointed and the offset of each peak from the midpoint is calculated [32]. This offset is used to calculate the delay in two signals and in return providing us with a 2D direction of arrival. Graham [33] used another method of the same analogy called de-depolarization of microphone signals. This process of cross spectacle analysis involves “diagonal-removal” employed with fixed sources but cannot be generalized for multiple sources. 

Moreover, this paper considers some important points like frequency range of interest, distance from the source, sound source type and noise map resolution while developing the array. Each of these points play major role in the directivity output of the array [28]. In order to ensure the noise map resolution and qualitative acoustics analysis, the experiments have always been performed in specially designed acoustic chambers. These chambers are either fully anechoic or semianechoic [34]. These chambers work on the absorption principle. They tend to absorb the sound energy coming from all directions. For this purpose, all the walls of the chamber are covered with absorption material foams [35]. The design of these foams itself is a study and it defines the absorption coefficient of the room. Most of the foams are pyramid- or wedge-shaped. For the perturbations from the floor, the floor is covered with foams and a wire mesh is spread over it for ease of experiments [36]. 

In recent years, many experimental techniques for the study of acoustic characteristics have been applied to VAWT. Mohamed [15] and Pearson [36,37] have shown in their research that higher tip speed ratios and higher RPMs result in higher noise spectrums. Moreover, the tonal noises are dominant in the lower frequency spectrum whereas broadband noises cover the whole spectrum. Their research shows one more important thing that most broadband noises are being generated from trailing edge interactions that cover vortex shedding noise and trailing edge noise. These predictions imply that the microphone techniques are still in development phase and have room for further research. This paper focuses on formulation of an in-house code for microphone array development and parametric study where TSR distance from the source is varied and results are later compared with a NORSONIC 128 MEMS acoustic camera and literature for validation of spectral results in this research linear array will be utilized to quantify the SPL levels at different axial locations and formulate the trend it follows. This trend is the basis for predicting the locations for wind turbine farms. The tonal and broadband range will be quantified and the effects of different tip speed ratios will be compared. The optimum tip speed ratio will be selected for the minimum noise levels so that a standard operating range for this particular turbine can be defined. A way forward will be devised in this research to suppress these acoustic sources. 

## 2. Design of Experiment

**Figure 1:**
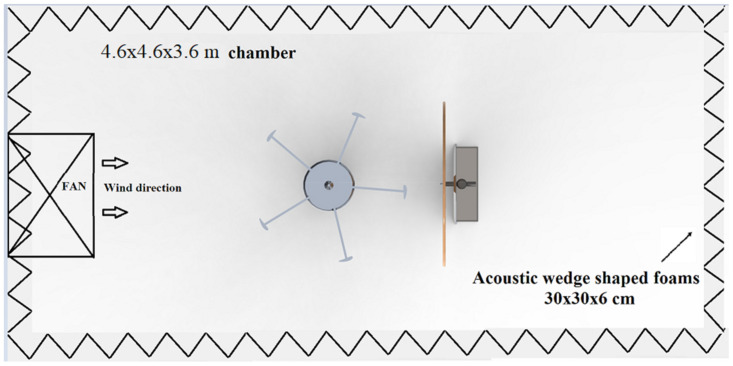
Experimental configuration of NUST Anechoic Chamber.

The experimental measurements were taken in an anechoic chamber in National University of Science and Technology (NUST), Islamabad. The schematic of experimental facility is shown in Figure 1.

**Figure 2:**
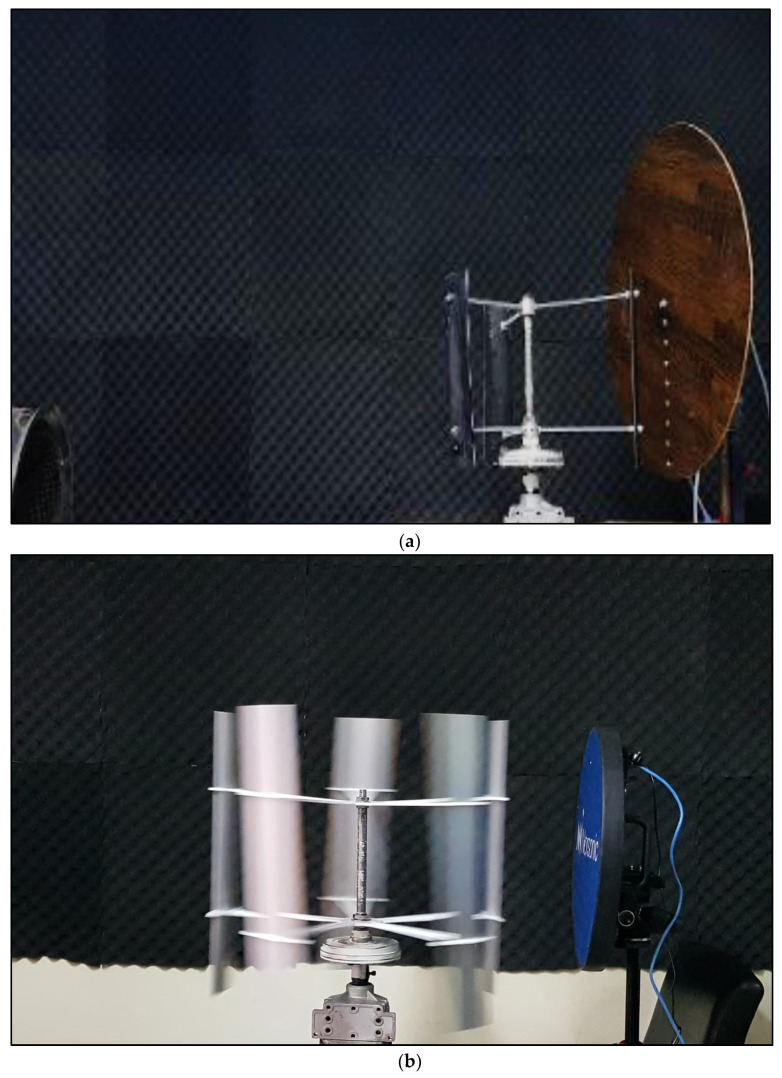
(**a**) Acoustic carpet sheet linear array (**b**) NORSONIC camera.

**Figure 3:**
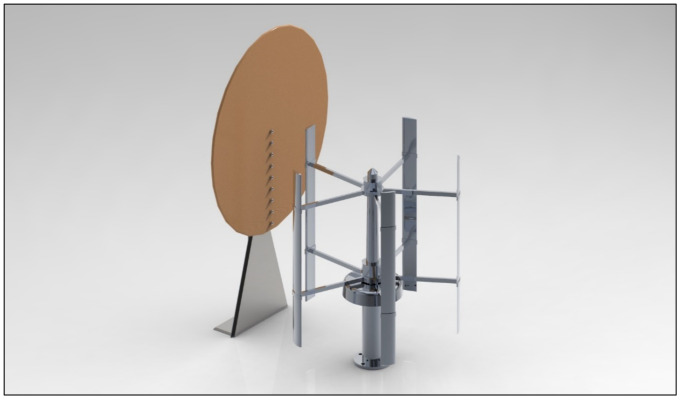
Linear microphone array placed in front of SAV-45 vertical axis wind turbine (VAWT).

NUST (National University of Science and Technology) anechoic chamber was utilized as measurement facility so that measurements were free of noise reflections from the walls and were more focused on source. The anechoic chamber was designed with an absorption coefficient of 0.6 for low frequencies. The anechoic chamber was a complete working environment with a cross section of (4.6 × 4.6 × 3.6) m^3^. The circular plate was made up of brown acoustic carpet giving it a nonreflective surface, thus saving the interference of measurements as shown in Figure 2. The microphone array installed in this chamber was mobile and could be placed anywhere in the chamber as shown in Figure 3.

**Figure 4:**
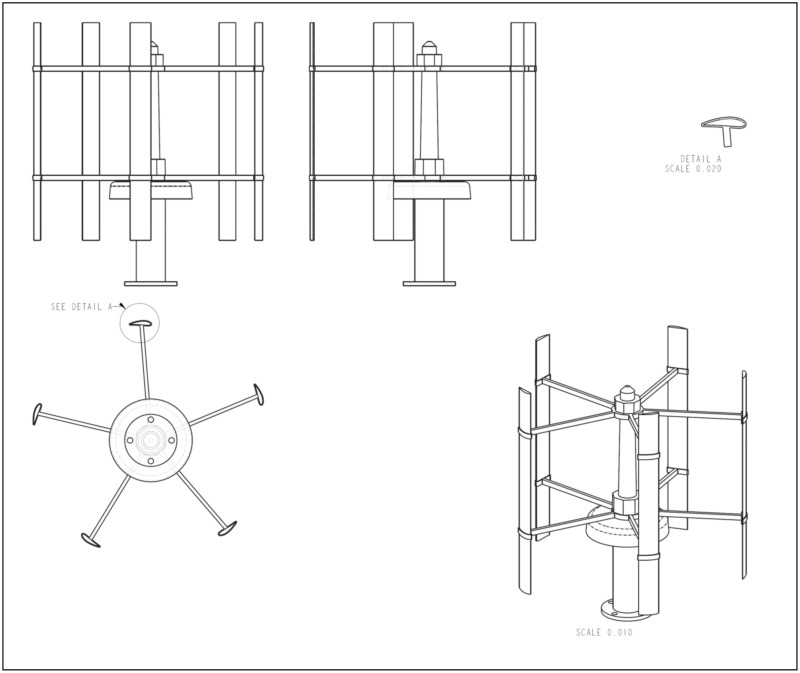
SAV-45 VAWT Schematic.

The source of noise in this experiment is a SAV-45 W VAWT. SAV-45 has five blades with a NACA 6418 airfoil. This wind turbine was chosen because of its good performance and low noise characteristics. The maximum attainable working RPM for this VAWT is 300. In order to attain a minimal working RPM of 250 for the rotor of SAV-45, a wind speed of 6–7 m/s was provided with the help of a ducted fan. The schematic of SAV-45 is shown in Figure 4. 

The details of the rotor of SAV-45 are illustrated in Table 1.

**Figure 5:**
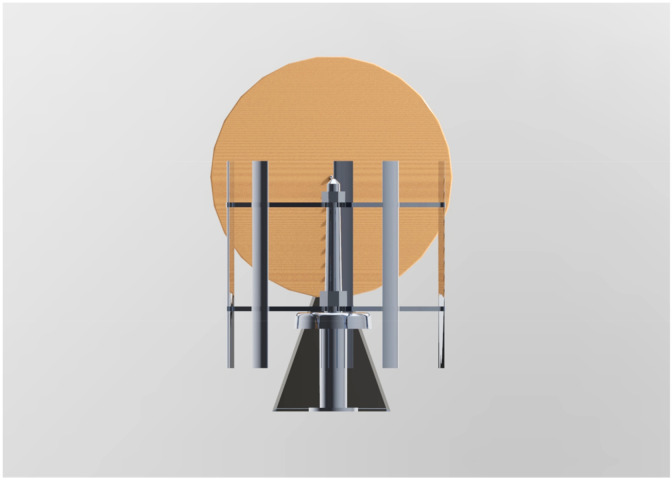
Microphone array mid rotor configuration.

In order to take acoustic measurements, eight (MAX4466) adjustable gain microphones were used to form a linear array. A linear array was developed using NI-DAQ 6009 and LabVIEW 2019. An in-house code was developed in LabVIEW for postprocessing of acquired raw signals. These eight microphones were placed linearly at midrotor configuration as shown in Figure 5. Each mic was 1.33 inches apart. The array was placed at three different axial locations from the source for different measurements. This array has a linear frequency response attribute. The frequency spectrum has a range of 8 Hz to 20 KHz. The height of the fourth microphone was approximately equal to the mid rotor height as shown in Figure 5.

In order to perform measurements at various TSRs, the duct’s air speed was maintained at a constant level and RPMs were varied using a variable speed DC motor mounted on the VAWT. RPM and air speed were traded for controlled TSRs. A variable DC supply was designed for controlled speed at five different voltages for future experiments at different TSR values. The details of rotation speed and corresponding TSRs are illustrated in Table 2. 

TSR values below 3 m/s wind speed were neglected because they are not in agreement with the working range of the turbine. Therefore, TSR values for only 4 m/s, 5 m/s and 6 m/s were tested and compared. The microphone signals were acquired at a sample rate of 6000 samples per second and the total test time was 1 s. For a 2D direction of arrival, cross-spectra between microphones was also acquired but this paper focuses mainly on quantification of noise levels from source. The cancellation of background noise was incorporated by taking measurements with and without the turbine model for comparison. Each mic signal was averaged for 10 fast Fourier transform (FFT) spectrums which means an average of 80 times that provided us with a better resolution. 

**Figure 6:**
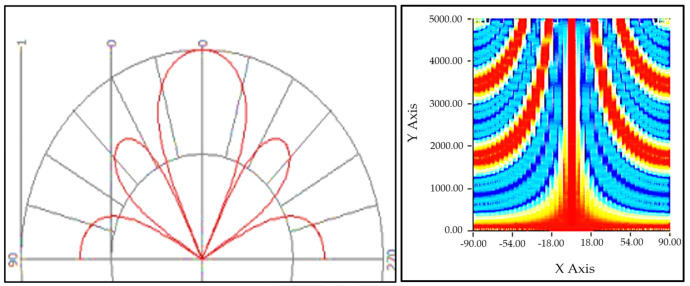
Broadside linear array resolution at 1 KHz.

In order to segregate the broadband and tonal features in results, a very high-resolution measurement was required. The directional resolution of the array was as per the linear structure, and the array was positioned to generate the best resolution at the outer 25% of the rotor when the array was in vertical position moving downwards. For linear arrays, the angular resolution decreases with the increasing frequency [38]. Our lowest frequency of interest was 40 Hz, so, as it was increased up to 1 kHz, the resolution decreased. Usually it is expected that the resolution is lower due to the coherence loss. Frontal position of the desired noise pickup was identified as zero degrees in the lobe diagram whereas the back side of the array was at 180°, and the sides point out to the space in between centered on 270° and 90°. This plot was normalized at the zero-degree response level and shown for our array in Figure 6.

**Figure 7:**
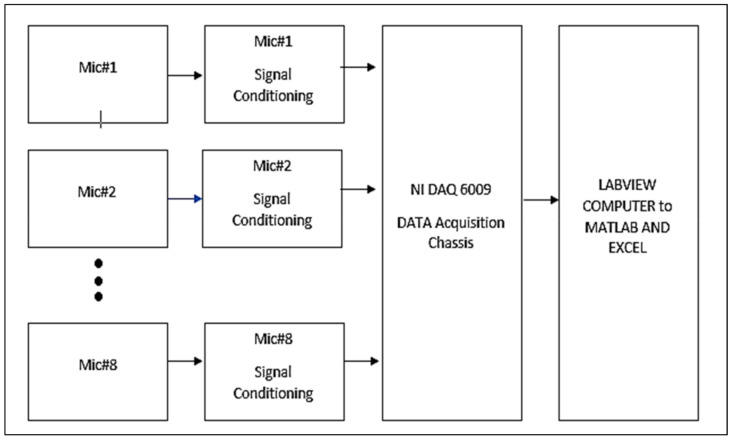
Array Hardware Topology.

**Figure 8:**
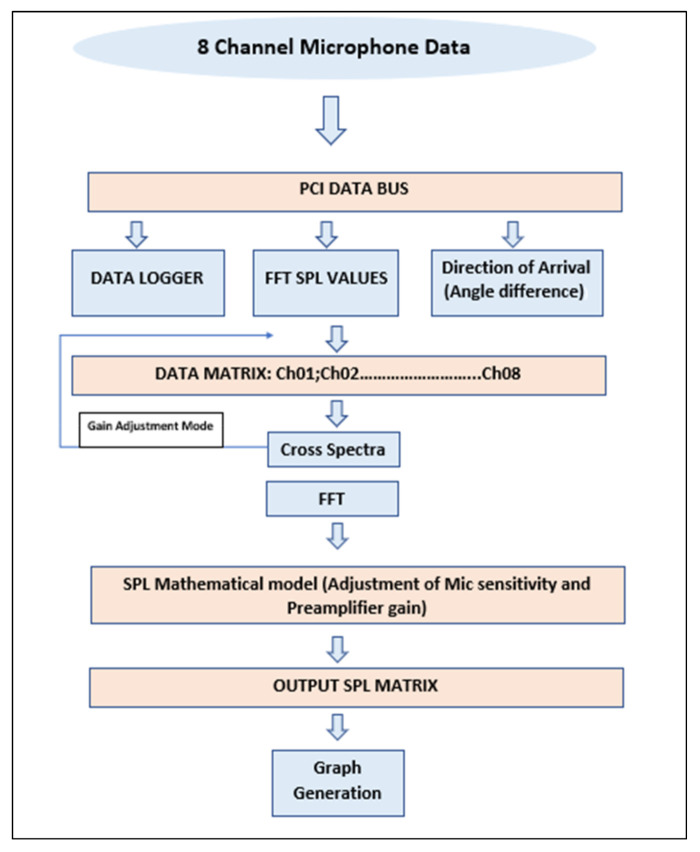
In-house code structure.

The LabVIEW in-house code developed for this array also allowed us to perform signal conditioning and delay correction for a 1.3 ms phase difference between each microphone. The in-house code was divided into three parts, i.e., test mode, gain equalization mode and gain adjustment mode. Each mode acquired RMS SPL values, implemented FFT and converted signals to dB scale while applying gain correction. Moreover, it compiled the data into a cluster and then wrote files to respective locations. In gain correction, one mic was chosen as reference and adjusted the gain of the rest of the mics in the software so that all microphones measure same background noise values. Each mode followed the same SPL algorithm apart from adjustments. The algorithm followed the structure presented in Figure 7. A weighing filter was incorporated manually in the results. The in-house code for this experiment is shown in Figure 8. This in-house code followed the general algorithm where DAQ-6009 acquired the raw voltage signals, logged them and applied fast Fourier transform on them. It converted the signal into SPL using formulas in the algorithm and wrote the spectrums in respective folders. 

## 3. Results and Discussion

**Figure 9:**
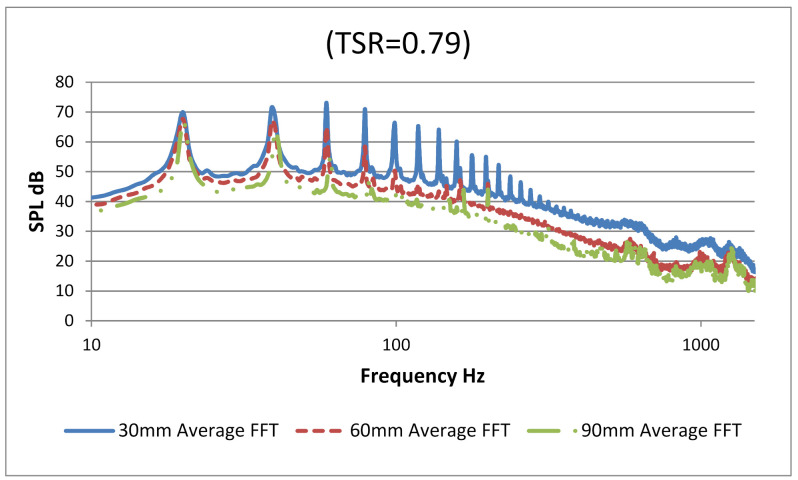
Frequency spectrum for five-bladed VAWT at axial distance of 30 mm, 60 mm and 90 mm from microphone array (TSR 0.79).

Figure 9 illustrates the frequency spectrum for TSR 0.79 for three axial distances, 30 mm, 60 mm, and 90 mm of microphone array from the source VAWT.

**Figure 10:**
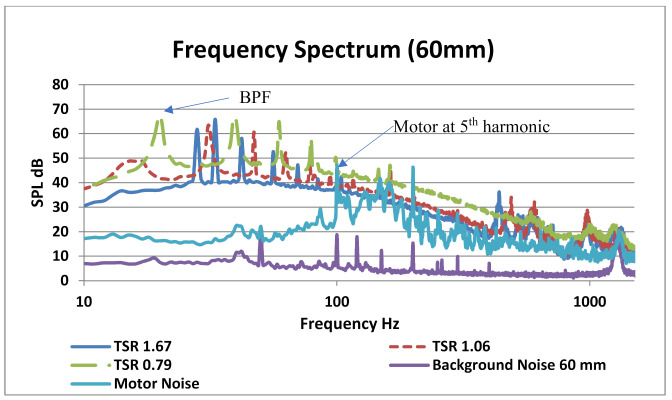
Frequency spectrum at three different TSR values at 60 mm axial location from source.

The measurement captured the amplitude of BPF for all three distances. The first harmonic clearly had highest BPF of 70 dB at 30 mm and lowest of 67 dB at 90 mm axial distance from source. The first five tonal peaks show the BPF (tonal characteristic) of five blades of the VAWT. However, from 100 Hz to 1500 Hz, downstream harmonics of tonal characteristics and broadband noises were observed. Generally, at this low tip speed ratio of 0.79, the noise characteristics were dominated by vortex interaction with blade noise which in turn generated the blade passing frequency and its higher harmonics. Since the contribution of higher harmonics is clear at this tip speed ratio of 0.79, which means that loading noise contributed largely into the (SPL average) value for this experiment. For tip speed ratios as low as 0.79, dynamic stall was likely to occur at the blades and, therefore, vortex shedding was strong in nature, contributing to higher harmonics of BPF [39]. Figure 10 shows the frequency spectrum for VAWT operated at three different TSR values. In this figure, background and motor noise was also incorporated. The background noises including the motor were subtracted from the overall spectrum. After the fifth blade’s passing frequency, the motor completed its one single revolution causing its tonal noise addition to the fifth blade harmonic. Figure 10 indicates that at lower tip speed ratios, there was likely to be a dynamic stall phenomenon due to which lower TSR values dominated at all harmonic regions.

**Figure 11:**
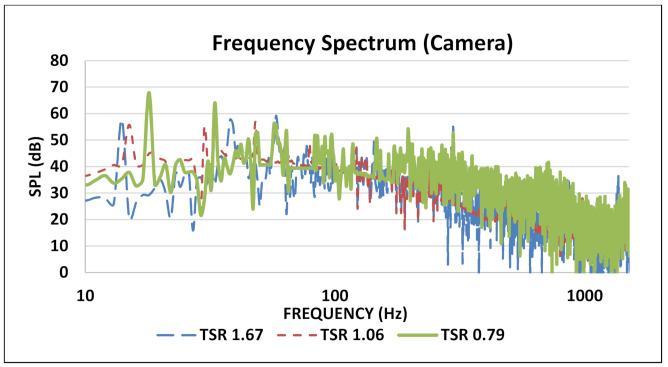
Frequency spectrum of SAV-45 using acoustic camera for three TSR values.

With the increase in TSR value, the dominant noise source in the blade traveled downstream of the rotor which was observed at the mic spectrum located at downstream position. Scheurich [40] showed that, as the TSR were increased, the vortices traveled downstream of the blade and were more likely to mix with shed vortices in wake flow. It can be seen that broadband spectrum is not disturbed too much by changing the TSR values. It indicates that the noise in the broadband region is more likely to originate from the self-stimulated regions like trailing edge noises. The tonal peaks indicate the leading edge blade passing frequency at different frequencies depicting that each blade has its own characteristic frequency depending on the wake flow and blade surface. Most of the vibrations occur in this region making for most part of the noise. Since total noise emission also involves background noise whereas structural noise is close to background spectrum therefore it can get neglected at times. In our case, the most significant spectral content is up to 90 Hz. The small peaks between the major harmonic peaks are the sound of bearings of the VAWT. Delay and sum beamforming was also applied using this array and the maximum direction of arrival using the phase difference was pointed towards the 45° and 135° of the trailing edge of the rotor, but since these microphones were not covering a broad spectrum having a smaller resolution, then focus was on quantification. In order to validate the results of the eight-channel microphone array, similar tests were performed using a NORSONIC 128 MEMS acoustic camera with a resolution of 16 bits per sample. Same boundary conditions were simulated during these tests and the results were compared. Figure 11 illustrates the frequency spectrum of SAV-45 VAWT using the NORSONIC acoustic camera. The trend followed by SPL values against frequency was similar to that of microphone array developed originally.

**Figure 12:**
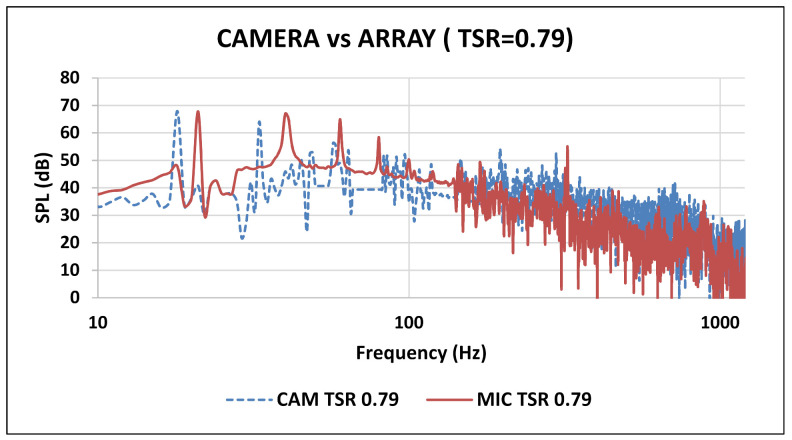
Frequency spectrum (NORSONIC camera—blue) and (microphone array—red).

The first harmonic BPF occurred around 18 Hz for 0.79 TSR, which falls under the tolerance range for the microphone array that is also the same. The comparison between the acoustic camera and microphone array developed for TSR value 0.79 is shown in Figure 12.

**Figure 13:**
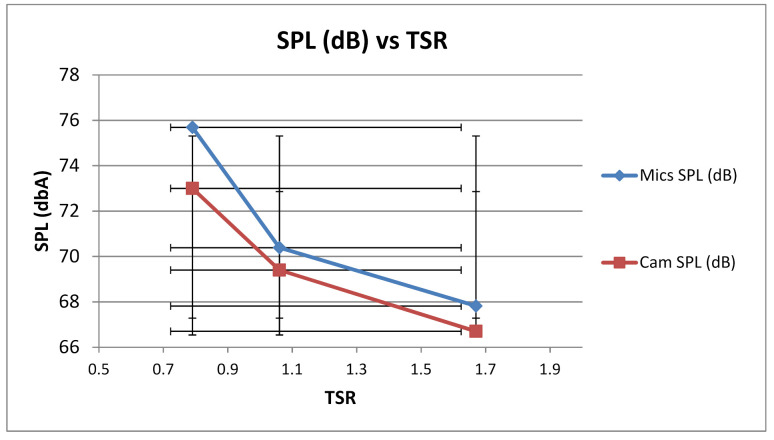
Error quantification spectrum for linear array compared to NORSONIC Camera.

While comparing the results of acoustic array and NORSONIC camera, it is evident that the general trend for tonal and broadband characteristics were in qualitative agreement with each other. It should be noted that results were compared at 60 mm axial distance. However, there was a slight disagreement in quantification of the SPL spectrum, which gave the dB margin error for tonal characteristics in our developed array. The averaged SPL values for all three TSR’s were compared and it provided 3 dB margin at low TSR and 1 dB margin at high TSR value as shown in Figure 13. It analyzed the results from literature, and provided the conclusion that 128 MEMS are more accurate and cover a broad spectrum for larger sources due to high resolution as compared to an eight-microphone linear array. This limits the scope of eight-microphone array to small rotating or static noise sources.

**Figure 14:**
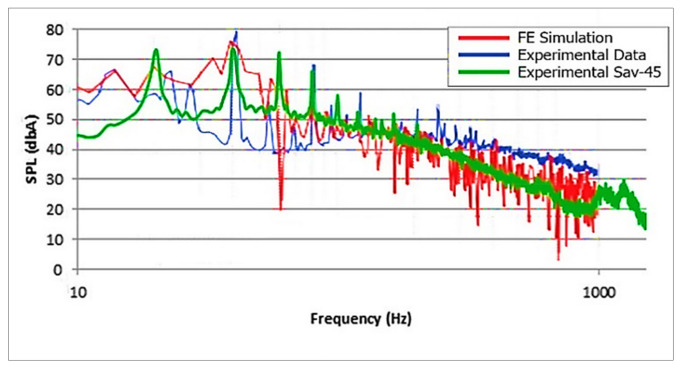
Data validation. Red–CFD simulation; blue–Weber’s experiment; green–SAV-45 array.

Moreover, the results gathered from the microphone array were compared with the work done by Weber [3]. Considering the design of Weber’s experiment, it is similar to this experiment. Weber used NACA 0018, a three-bladed VAWT at TSR 0.5 which is compared with TSR = 0.79. The data points gathered from Weber’s work and axis were equated and normalized on scale in order to compare both results, as shown in Figure 14.

Figure 14 illustrates the matching trend of SAV-45 VAWT and Weber’s VAWT. The first harmonics had different BPF amplitudes because of the different solidity and number of blades of VAWT. CFD simulation of Weber’s experiment and experimental results of the NORSONIC acoustic camera both exhibited lower values of harmonics in broadband spectrum. It was because the directivity of the camera and simulation was far better than the microphone array and covered a larger azimuth angle, thus generating a broader spectrum.

**Figure 15:**
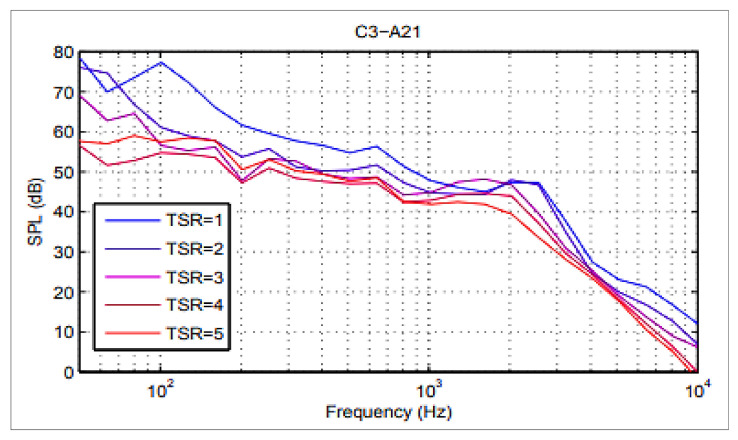
Data validation with Pearson three-bladed VAWT (TSR Variation).

Pearson and Graham also performed a similar experiment where they showed that with increasing TSR then the SPL decreases and an optimum TSR has to be attained for better acoustic performance and all those results were also complementing this research [37]. The TSR trend for their work is shown in Figure 15, following the same trend as this research.

## 4. Conclusions

Experimental measurements were performed on a camber-bladed five-blade vertical axis wind turbine. The frequency spectrum for TSR 0.79, 1.06 and 1.67 followed the same trend for tonal and broadband region as for the results of the NORSONIC acoustic camera. There was slight disagreement in quantitative comparison for both pieces of equipment. The NORSONIC camera was more accurate for the broad range measurements due to its 128 MEMS directivity whereas for small region measurements the linear microphone array was more accurate and cost effective. It was deduced from the results that, at lower TSR values, dynamic stall was evident causing vortex shedding and in return higher SPL values were generated. This dynamic stall noise occurrence could be improved by operating at optimum TSR value of 1.06 in this case and providing a preset pitch angle [41]. Another important agreement between the camera and linear array was that both trends followed the 1/r law for increasing distance from the source providing us a way forward for designing wind farm architecture. Moreover, the broadband spectrum was disturbed on a negligible level by changing the TSR values, therefore, broadband noises originated from self-stimulated regions such as trailing edge noises. The data validation of this experiment with similar design of experiment performed by Weber provides a way forward for further research with the developed algorithm on reduction of noise mechanism in cambered bladed turbines using serrations, etc.

Moreover, the linear array with vertical alignment can quantify noise sources on the blades for a wide-ranging area around the vertical positions of the rotor. A linear array, when placed horizontally and linearly; can quantify only half of the blade for a whole revolution. Comparing the resolution of our linear array and other circular arrays, we deduce that the linear array requires a lower number of microphones and a smaller length to attain an optimum resolution for a small area measurement like our vertical axis wind turbine.

## 5. Highlights

1. Design and development of an eight-microphone linear acoustic array.
2. Preparation of an anechoic chamber facility for acoustic experiments.
3. Preparation of a design of experiment including motor-controlled VAWT.
4. Data acquisition of spectral matrices for linear microphone array and NORSONIC acoustic camera.
5. Validation of linear array results with NORSONIC camera and literature.

## 6. Future Recommendation

1. Upgrading of microphone array to 3D source localization acoustic camera and identifying discrete noise sources during dynamic stall can provide very useful information for design changes required.
2. Including the nonintrusive measurements like LDA/PDA and PIV/Hotwire techniques into these measurements to better understand the noise sources through aerodynamic perspectives [38].
3. The effect of rotor solidity can be taken into consideration and the type of airfoil and the number of blades can be changed on the same VAWT to find a way through to minimize the noise sources [40].
4. After incorporating a properly validated microphone array, an experiment must be performed on VAWT installed in series in farms to see the effect of the wake of one VAWT on another.

## Figures and Tables

**Table 1:** SAV-45 VAWT specifications.

Diameter	R-Length	Blade	Swept Area	Weight
560 mm	525 mm	5 Aluminum Alloy	0.3 m2	7.5 kg

**Table 2:** Setup of parameters.

Turbine RPM (rev/min)	Wind Speed (m/s) (approx.)	TSR
130	7	0.53
163	6	0.79
183	5	1.06
235	4	1.67

## Nomenclature

TSR	Tip Speed Ratio
SPL	Sound Pressure Level
BTV	Blade Tip Vortex
HAWT	Horizontal Axis Wind Turbine
LES	Large eddy simulation
